# The Integral Role of CBOCs in Rural Healthcare: Promises and Challenges

**DOI:** 10.1093/geroni/igab046.455

**Published:** 2021-12-17

**Authors:** Camilla Pimentel, Kathryn Nearing, Laura Kernan, Eileen Dryden, Lauren Moo

**Affiliations:** 1 VA Bedford Healthcare System, Bedford, Massachusetts, United States; 2 Veterans Health Administration, Denver, Colorado, United States; 3 Veterans Health Administration, Bedford, Massachusetts, United States; 4 VA Bedford Health Care System, Bedford, Massachusetts, United States

## Abstract

Community-based outpatient clinics are critical to extending the geographic reach of VA’s healthcare delivery system. Nationwide, 733 CBOCs provide outpatient care to nearly half of the VA’s patient population. The 13 rural CBOCs in the study sample provide outpatient primary care, mental health care, and a limited number of specialty care services. Located 1–3.5 hours away from their closest VA Medical Center, these CBOCs have a wide—sometimes interstate—service catchment area. To effectively serve increasingly older and medically complex patient populations, they rely heavily on partnerships with larger VA Medical Centers and local community providers for inpatient, residential, and additional outpatient services. CBOCs experience myriad staffing challenges, including staff turnover, “access providers” working at multiple CBOCs, and highly variable training in rural health and geriatrics. While some CBOCs have robust telehealth offerings, others cannot currently grow their telehealth capacity owing to constraints in clinic space and provider schedules.

